# Development of ^11^C-Labeled ASEM Analogues for the Detection of Neuronal Nicotinic Acetylcholine Receptors (α7-nAChR)

**DOI:** 10.1021/acschemneuro.1c00730

**Published:** 2022-01-12

**Authors:** Sangram Nag, Patricia Miranda-Azpiazu, Zhisheng Jia, Prodip Datta, Ryosuke Arakawa, Mohammad Mahdi Moein, Zhou Yang, Yaoquan Tu, Laetitia Lemoine, Hans Ågren, Agneta Nordberg, Bengt Långström, Christer Halldin

**Affiliations:** † Department of Clinical Neuroscience, Centre for Psychiatry Research, Karolinska Institutet and Stockholm County Council, 171 76 Stockholm, Sweden; ‡ Department of Physics and Astronomy, Uppsala University, 751 20 Uppsala, Sweden; § Division of Theoretical Chemistry and Biology, Royal Institute of Technology (KTH), 11428 Stockholm, Sweden; ∥ Department of Neurobiology, Care Sciences and Society, Karolinska Institutet, 141 52 Stockholm Sweden; ⊥ Department of Chemistry, Uppsala University, 75123 Uppsala, Sweden; # Theme Aging, Karolinska University Hospital, 141 52 Stockholm, Sweden

**Keywords:** α7-nAChR, PET, non-human primate, autoradiography, radiometabolites, in vivo, in vitro

## Abstract

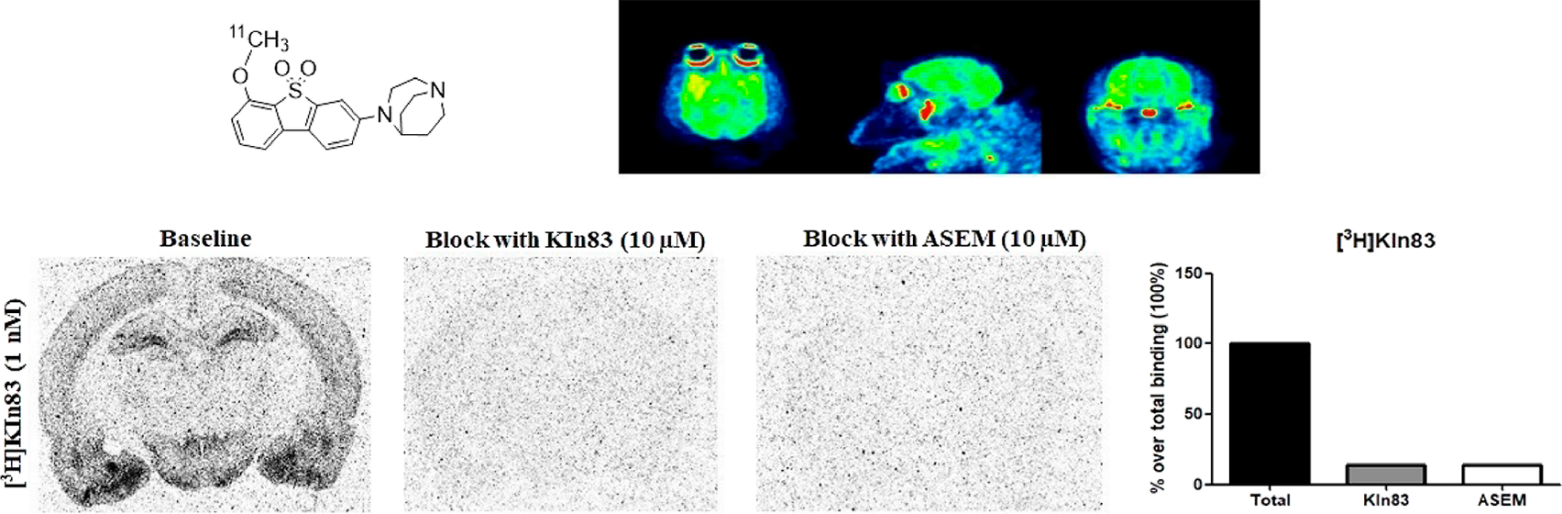

The homo-pentameric alpha 7 receptor is one of the major types of neuronal nicotinic acetylcholine receptors (α7-nAChRs) related to cognition, memory formation, and attention processing. The mapping of α7-nAChRs by PET pulls a lot of attention to realize the mechanism and development of CNS diseases such as AD, PD, and schizophrenia. Several PET radioligands have been explored for the detection of the α7-nAChR. ^18^F-ASEM is the most functional for *in vivo* quantification of α7-nAChRs in the human brain. The first aim of this study was to initially use results from in silico and machine learning techniques to prescreen and predict the binding energy and other properties of ASEM analogues and to interpret these properties in terms of atomic structures using ^18^F-ASEM as a lead structure, and second, to label some selected candidates with carbon-11/hydrogen-3 (^11^C/^3^H) and to evaluate the binding properties *in vitro* and *in vivo* using the labeled candidates. In silico predictions are obtained from perturbation free-energy calculations preceded by molecular docking, molecular dynamics, and metadynamics simulations. Machine learning techniques have been applied for the BBB and P-gp-binding properties. Six analogues of ASEM were labeled with ^11^C, and three of them were additionally labeled with ^3^H. Binding properties were further evaluated using autoradiography (ARG) and PET measurements in non-human primates (NHPs). Radiometabolites were measured in NHP plasma. All six compounds were successfully synthesized. Evaluation with ARG showed that ^11^C-Kln83 was preferably binding to the α7-nAChR. Competition studies showed that 80% of the total binding was displaced. Further ARG studies using ^3^H-KIn-83 replicated the preliminary results. In the NHP PET study, the distribution pattern of ^11^C-KIn-83 was similar to other α7 nAChR PET tracers. The brain uptake was relatively low and increased by the administration of tariquidar, indicating a substrate of P-gp. The ASEM blocking study showed that ^11^C-KIn-83 specifically binds to α7 nAChRs. Preliminary *in vitro* evaluation of KIn-83 by ARG with both ^11^C and ^3^H and *in vivo* evaluation in NHP showed favorable properties for selectively imaging α7-nAChRs, despite a relatively low brain uptake.

## Introduction

Nicotinic acetylcholine receptors (nAChRs) are receptor polypeptides that respond to the neurotransmitter acetylcholine. Based on the compositions of the subunits, nAChRs can be divided into two different types, such as muscle and neuronal nAChRs. The neuronal nAChR subtypes again varied in homomeric or heteromeric combinations of 12 different nicotinic receptor subunits, α2−α10 and β2−β4.^1,2^

Homomeric α7 nAChRs (α7 nAChRs), mainly expressed in the CNS and spinal cord, are distinguished from neuronal heteromeric nAChRs by their high-affinity binding to α-bungarotoxin. For decades, it was assumed that neuronal nAChRs are exclusively expressed on neurons. Nevertheless, the recent research has shown that functional nAChR responses can be found in non-excitable cells, including microglia^3,4^ and astrocytes.^5^ Thus, the α7 nAChRs are involved in several cognitive and physiologic processes; its appearance levels and patterns change in neurodegenerative and psychiatric diseases, such as Parkinson’s disease (PD), Alzheimer’s disease (AD), or schizophrenia, which makes it a significant drug target.^6−10^

**Figure 1:**
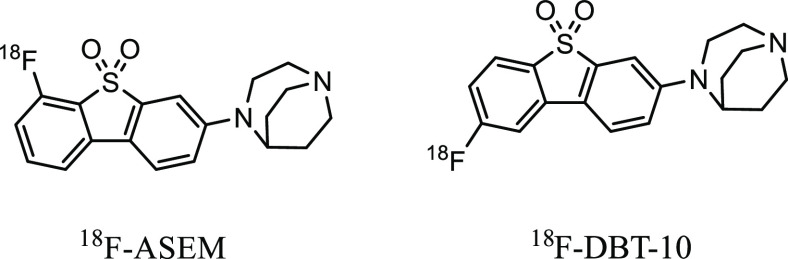
Structures of radioligands, ^18^F-ASEM and ^18^F-DBT-10.

Positron emission tomography (PET), a sensitive and non-invasive molecular imaging technique, has been successfully utilized in visualizing the localization of different targets in the brain^11,12^ such as α7 nAChRs.^13^^11^C-CHIBA-1001 was the first PET radioligand to image α7 nAChRs in the human brain, which showed reduced specificity for α_7_ nAChRs and high nonspecific uptake.^14^ Later on, ^18^F-ASEM and [^18^F]DBT-10, corresponding two isomers based on the dibenzothiophene skeleton (Figure 1), which only differs in the position of the fluoro substituent,^7^ were characterized both *in vitro* and *in vivo*.^13,15−18^ Recent studies using ^18^F-ASEM and ^18^F-DBT-10 further stated the suitability of the tracers, showing high and reversible brain uptake with a regional binding pattern consistent with the distribution of α7 nAChR receptors in the non-human primate (NHP) brain.^18^ [^125^I]Iodo-ASEM indicated that provides sensitive and selective imaging of α7 nAChR *in vitro*, with better signal-to-noise ratio than previously developed tracers.^19^ Human PET studies^10,13,19,20^ suggested the general applicability of ^18^F-ASEM-binding properties, and interpretation of novel α7 nAChR tracers might be complicated by the fact that α7 subunits can form heteromeric receptors together with other subunits, such as β2;^9,19,21^ however, it remains unclear how this affects to the selectivity of the radiotracer binding. Development of more selective radioligands is significant for describing the binding properties and occupancy of molecules targeting the receptor.^19^

Modern in silico techniques that have been applied encompass the most important aspects of tracer prediction of ASEM and its analogues, like the structural sources of binding, location of multiple binding sites, the binding strengths, transition-state barriers, and kinetics and dynamical factors of the tracer protein interactions.^22−24^ The hierarchical multi-level approaches represent different levels of rigour and efficiency, involving molecular docking, implicit solvent models, metadynamics, and free-energy perturbation calculations. In particular, protein structures based on newly developed cryomicroscopy have made it possible to go into a considerable depth in the evaluation of the atomic origin of the binding.

Therefore, our aims of this project were (i) to use results from in silico and machine learning techniques to prescreen ASEM analogues, (ii) to explore and develop efficient synthetic methods for labeling the selected candidates with ^11^C and ^3^H, (iii) to evaluate the *in vitro* autoradiography (ARG) in the *postmortem* rat/human brain, and (iv) to study the *in vivo* characteristics by PET measurements in NHPs, including radiometabolite analysis in plasma.

## Results and Discussion

### In Silico and Machine Learning Data

Results from the tracer interaction with α7-nAChR using the structure-based in silico rational strategy and ligand-based machine learning methods are given in Table 2. The data recapitulated in the table are an excerpt from a larger tabulation given including 14 compounds (except for the newly computed Kd rates).^22^ The differential binding energies are given as relative binging free energies with respect to the ASEM compound. Results for the residence time of the tracer derives from kinetics of the unbinding process, as obtained from potential scaled MD and metadynamics simulations.^2^ Based on the structure information obtained, the binding free energy and residence time in the pocket are given for the ASEM analogue series. Besides, using machine learning, we have also analyzed the blood–brain barrier (BBB) penetration and P-gp protein-binding properties. Thus, from the rational modeling, we predict free binding energies (ΔΔ*G*) with respect to a reference compound ASEM and residence times for the tracer in the α7-nAChR pockets; from machine learning, we predict log *P*—the solubility, the plasma protein binding, the BBB capability, and the P-gp substrate-binding strength.

**Table 1:** Optimization of Radiosynthesis

radioligand	precursor	amount of precursor (mg)	alkylating agent	solvent (mL)	base (mg)	reaction temp (°C)	reaction time (min)
[^11^C]KIn-74	PRE-4	1.0	^11^C-CH_3_I	DMF (0.5 mL)	CsCO_2_ (5.0 mg)	80	4
[^11^C]KIn-75	PRE-2	0.5	^11^C-CH_3_I	DMSO (0.5 mL)	KOH (5.0 mg)	90	5
[^11^C]KIn-77	Kin-75	1.0	^11^C-CH_3_I	DMSO (0.5 mL)	NaOH (5.0 mg	90	5
[^11^C]KIn-83	PRE-3	0.5	^11^C-CH_3_I	DMF (0.5 mL)	CsCO_2_ (5.0 mg)	80	4
[^11^C]KIn-84	PRE-1	1.5–2.0	^11^C-CH_3_I	DMSO (0.5 mL)	KOH (7.0 mg)	80	3
[^11^C]KIn-85	KIn-84	1.0	^11^C-CH_3_I	DMSO (0.5 mL)	KOH (5.0 mg)	90	5
^3^H-KIn-74	PRE-4	1.0–2.0	^3^H-CH_3_I	DMSO (0.3 mL)	KOH (7.0 mg)	90	30
^3^H-KIn-83	PRE-3	1.0–2.0	^3^H-CH_3_I	DMSO (0.3 mL)	KOH (7.0 mg)	90	30
^3^H-KIn-84	PRE-1	1.0	^3^H-CH_3_I	DMF (0.3 mL)	CsCO_2_ (5.0 mg)	90	30

**Table 2:**
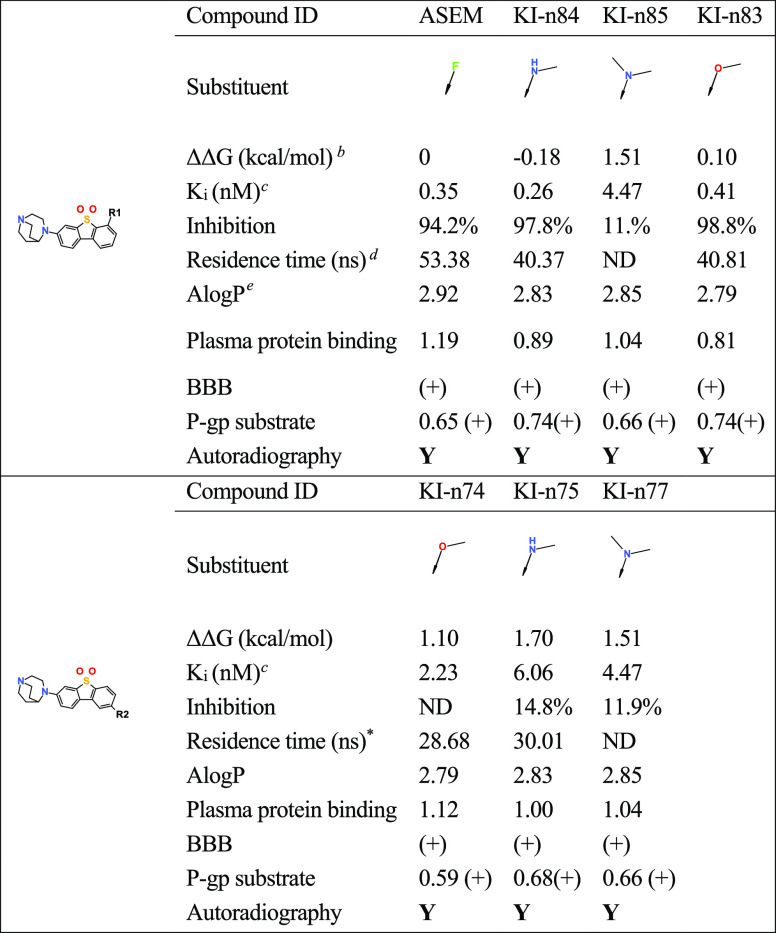
PET Tracer Data for α7-nAChR Using Rational Tracer Design and Machine Learning Methods^a^

a Data excerpt from tabulation given in ref FEP.

b (1) The binding free energy is calculated using FEP+ of Schrodinger.^23^ (2) The relative free energy is calculated with ASEM as the reference. Smaller is better.^24^

c The *K*_i_ value is calculated using Δ*G* = *–RT*ln(*K*/*K*_0_). The *K*_i_ value of ASEM was reported previously.^20^

d The residence time is calculated with potential scaled MD simulations using Gromacs.

e The physiochemical properties are predicted by machine learning methods based on cheminformatics using Python, sklearn, and rdkit.

Our previous studies^22,23^ have predicted the binding mode of ASEM and its analogues. Docking and FEP (Free Energy Perturbation) calculation show that large substitution at R2 position will decrease the binding affinity of the compound; in this study, we mainly focused on the compound with substitutions at R1 position and small-size substitution at R2 position. Molecular docking shows that KIn-83 fits to the same binding pocket of a7-nAChR for ASEM, indicating that KIn-83 and ASEM have the same binding site. This agrees with the ARG study, showing that 3H-KIn-83 was completely blocked by ASEM. Free-energy calculation shows that KIn-83 has a similar binding affinity with ASEM, while potential scaled MD shows that the unbinding rate of KIn-83 is faster than ASEM. Therefore, KIn-83 has a similar thermodynamic property to ASEM but a somewhat different kinetics property compared to ASEM. Machine learning predicted that KI-n83 is a P-gp substrate and can cross the BBB. This agrees with the results.

For the compounds substituted at positions R1 and R2, we can see that when ΔΔ*G* relative to ASEM is greater than 1 kcal/mol (e.g., KI-n74, KI-n75, KI-n77, and KI-n85), the compounds show low inhibition (inhibition <15%). When ΔΔ*G* is similar to ASEM (e.g., KI-n83, ΔΔ*G* = 0.1 kcal/mol) or lower than ASEM (e.g., KI-n84, ΔΔ*G* = −0.18 kcal/mol), the compounds show high inhibition (inhibition > 98%) (Table 2). This indicates that our theoretical calculations can predict the experimental results quite well. The residence times we calculated have a correlation with the inhibition rate although the few numbers of comparisons do not make it possible to settle the precise nature of the correlation. The two compounds with high affinity, KI-n83 and KI-n84, also have longer residence time (>40 ns), while the residence time of the compound with low affinity is shorter (<30 ns). The residence times of KI-n83 and KI-n84 are shorter than that for ASEM, so the two compounds could, with advantage, be used for a PET assay study. The calculated log *P* of the compounds are all below 3, indicating that they have good solubility. From the prediction of machine learning, the compounds can pass through the BBB+ and are the substrates of P-gp protein (P-gp+). Therefore, these compounds are assumed to be potentially good tracers for the CNS applications. Among them, KI-n83 and KI-n84 can bind α7-nAChR with high affinity, and with the advantage that the theoretical residence times are shorter than that of ASEM. We have performed ARG experiments (ARG+) on ASEM, KI-n75, KI-n83, KI-n84, and KI-n74 compounds.

We have furthermore predicted the *K*_i_ concentration values for the compounds in Table 2. Here, we applied the rate equation *K*_i_ = exp(−Δ*G*/*RT*) where Δ*G* is the free energy, *R* is the ideal gas constant, and *T* is the (room) temperature. The *K*_i_ value for a particular tracer is then obtained from Δ*G* = *–RT*ln(*K*/*K*_0_) where *K*_0_ is the value for the reference compound ASEM, as obtained from the previously published literature.^20^ We see that the predicted *K*_i_ values are well below 1 nm for KI-n83, KI-n84, and KI-n85, as for ASEM, while they are well above this limit for KI-n74, KI-n75, and KI-n77, indicating a clear preference for the former set of compounds.

### Radiochemistry

Cyclotron target produced ^11^C-CH_4_ and was utilized for the production of ^11^C-CH_3_I. The total time for radiosynthesis including purification and formulation of all six radioligands was about 30 min. The one-step ^11^C-methylation for all ligands was highly reproducible, and it produced 550–1600 MBq of the pure product for the specific radioligand following irradiation of the target with a beam current of 35 μA for 15–20 min. Molar activity (MA) of all six radioligands were > 165 GBq/μmol. The radiochemical purity was >99% at end of synthesis (EOS), and the identity of the radioligand was confirmed by the co-injection of the radioligand with an authentic standard by radio-high-performance liquid chromatography (HPLC). The formulated solution of the respective radioligand was found to be pure more than 99% for up to 1 h.

**Figure 2:**
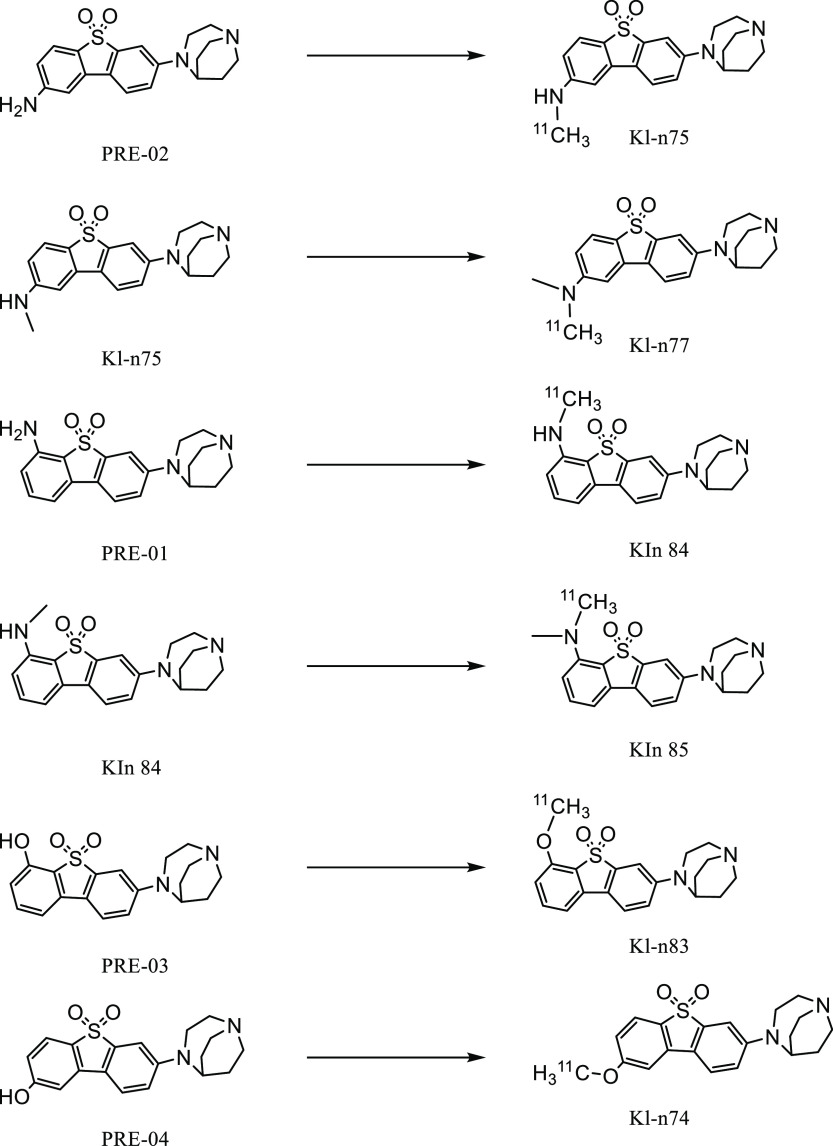
Radiosynthesis of ^11^C-KIn-74, ^11^C-KIn-75, ^11^C-KIn-77, ^11^C-KIn-83, ^11^C-KIn-84, and ^11^C-KIn-85.

A rapid and effective one-step radiosynthesis of six novel radioligands, ^11^C-KIn-74, ^11^C-KIn-75, ^11^C-KIn-77, ^11^C-KIn-83, ^11^C-KIn-84, and ^11^C-KIn-85 (Figure 2), was developed with high-yield purity and MA. Selective N- or O-methylation of the corresponding precursor was achieved using ^11^C-CH_3_I as the alkylating agent. Several different bases, such as NaOH, KOH, NaH, Na_2_CO_3_, and CsCO_3_, and different reaction solvents, such as acetone, DMSO, DMF, and MeOH, were explored to develop the optimal radiosynthesis conditions. For all the radiosynthesis, it was found that the combination of ^11^C-CH_3_I as the alkylating agent and DMF/DMSO with specific base at ambient temperatures were suitable for an optimal radiochemical yield. The final desired product was eluted from the solid-phase extraction (SPE) cartridge using ethanol and formulated into phosphate-buffered solution (PBS) containing less than 10% ethanol.

**Figure 3:**
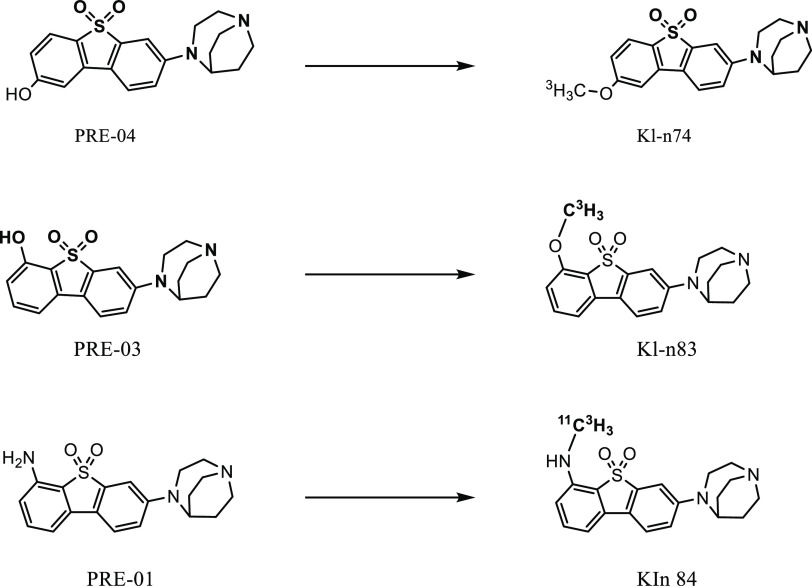
Radiosynthesis of ^3^H-KIn-74, ^3^H-KIn-83, and ^3^H-KIn-84.

^3^H-Methyl Iodide (^3^H-CH_3_I) was used to synthesize ^3^H-KIn-74, ^3^H-KIn-83, and ^3^H-KIn-84 via one step *N*-methylation/*O*-methylation of the corresponding precursor (Figure 3). The obtained MA of all three compounds was >1 GBq/μmol, and the radiochemical purity was >96% up to several months after radiosynthesis when stored at −20 °C.

### Autoradiography

Binding selectivity of all six compounds for α7-nAChR was evaluated by ARG, as a preliminary screening (data not shown). Taking the library concept to a radiochemical environment is a promising approach toward experimental tracer development for PET studies.

**Figure 4:**
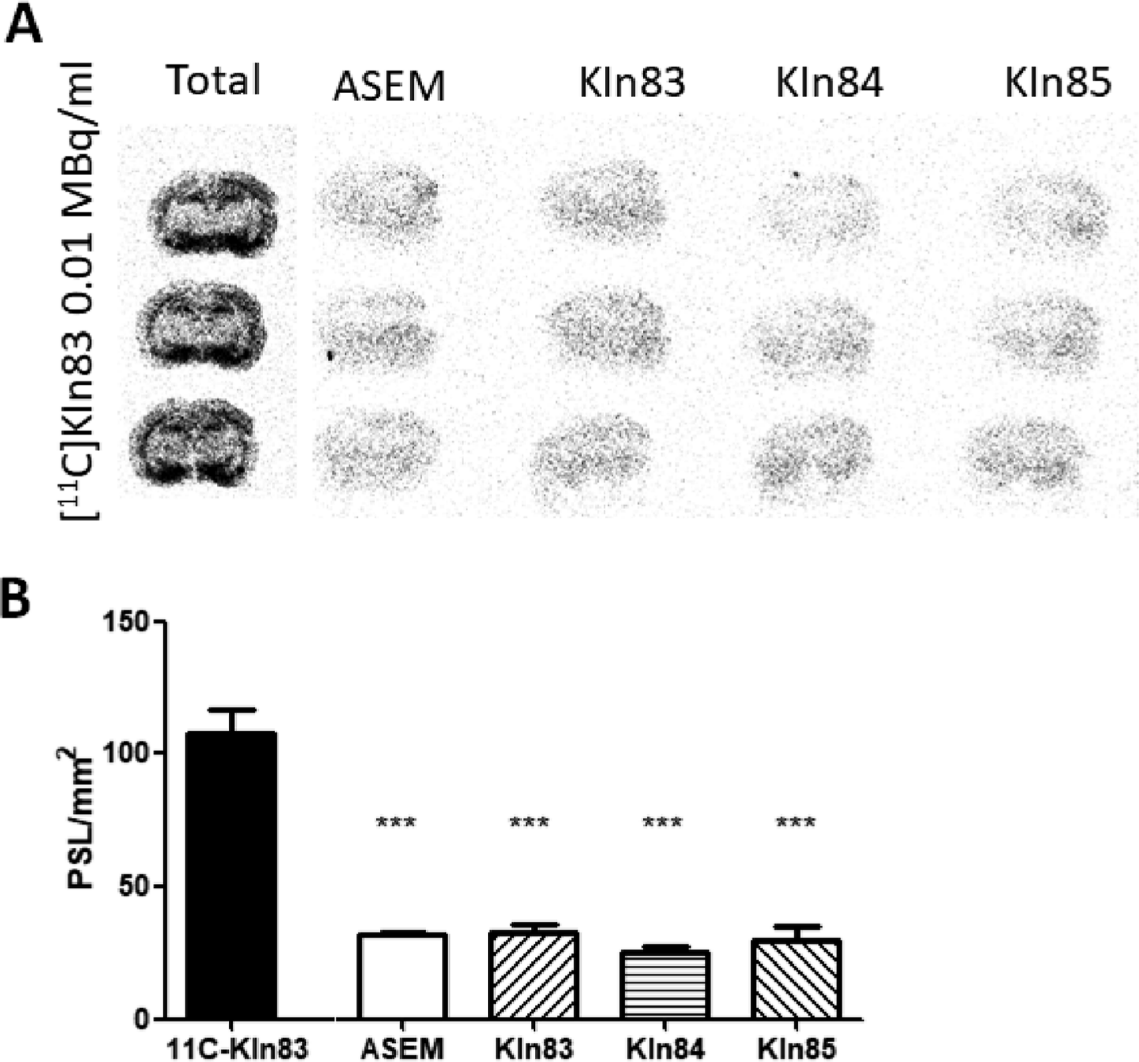
(A) Autoradiogram obtained showing total binding obtained with ^11^C-KIn-83 (0.01 MBq/mL) and non-specific binding using different blockers at 10 μM (ASEM, KIn-83, KIn-84, and KIn-85) in rat at the hippocampus level section. (B) Quantification of ^11^C-KIn-83 (0.01 MBq/mL) total and non-specific binding (expressed in PSL/mm^2^).

Evaluation with ARG showed that ^11^C-KIn-83 (0.01 MBq/mL) binds to α7-nAChR in the rat brain, showing the best signal to the brain regions containing the highest density of α7 nicotinic receptors; hippocampus, hypothalamus, and the cerebral cortex (Figure 4A). ARG competition studies showed that 80% of the total binding exerted by ^11^C-KIn-83 in rat brain tissue was displaced by adding 10 μM of ASEM and unlabeled KIn-83 (Figure 4B). KIn-84 and KIn85 (other ASEM analogues sharing the same binding sites for α7-nAChR) were also able to displace this binding to the same extent (Figure 4C).

**Figure 5:**
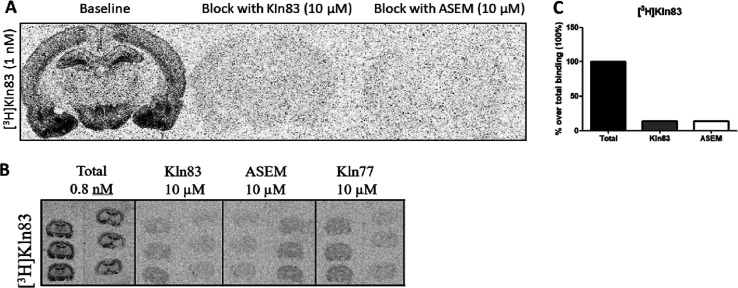
(A) Autoradiograms showing the total and non-specific binding (blocked with the homologous cold compound and ASEM at 10 μM) obtained in rat when using ^3^H-KIn-83 at a 1 nM concentration. (B) Autoradiograms showing the total and non-specific binding (blocked with the homologous cold compound (10 μM), ASEM (10 μM), KIn-77 (10 μM), and nicotine (100 μM) obtained in rat when using ^3^H-KIn-83 at a 0.8 nM concentration. (C) Quantification of total and nonspecific binding for ^3^H-KIn-83 expressed as percentage over total binding (100%).

KIn-83 was then tritiated in order to get a higher image resolution and the possibility of quantifying the specific binding to each brain region, separately. Thus, further ARG studies were performed with the tritiated version of KIn-83 (^3^H-KIn-83), replicating the results obtained with ^11^C-KIn-83 using a low concentration of tracer (0.8–1 nM). As it is observed in Figure 5A,C, autoradiogram showed a high specific binding to the brain regions of interest, which was completely blocked by both unlabeled KIn-83 and ASEM (10 μM), suggesting that both compounds share the same binding sites for α7 nAChR. Figure 5B shows how unlabeled KIn-77 (10 μM) was also able to block ^11^C-KIn-83 to the same extent as both unlabeled KIn-83 and ASEM, in principle suggesting that other binding sites (apart of the one shared with ASEM) could also be targeted by KIn-83 for α7 nAChR.

**Figure 6:**
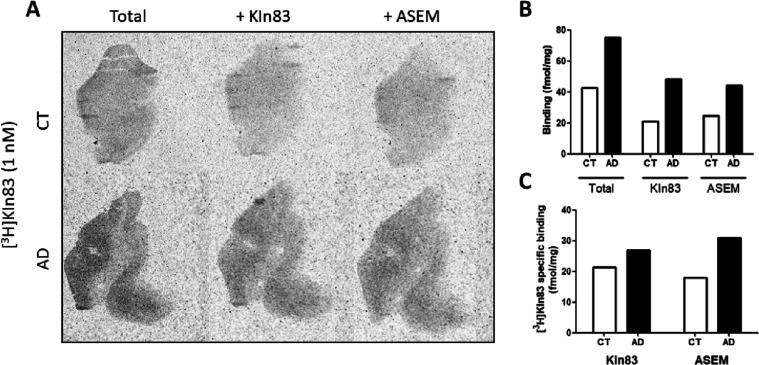
(A) Autoradiogram showing the total binding obtained using ^3^H-KIn-83 (1 nM) and non-specific binding (blocked with KIn-83 and ASEM at 10 μM) obtained in the temporal cortex of human tissue from a healthy control (CT) and an AD patient. (B) Quantification of total and non-specific binding for ^3^H-KIn-83 in control (white bars) and PD tissue (black bars) obtained when blocking with KIn-83 or ASEM. (C) Specific binding obtained blocking with KIn-83 and ASEM. Data are expressed in fmol/mg.

^11^C-KIn-83 (1 nM) was also tested with ARG using the human brain from a single AD case and a cognitive healthy control (CT), as depicted in Figure 6A. Figure 6B shows the total binding obtained in control tissue (around 40 fmol/mg) and the AD case (around 75 fmol/mg). However, the nonspecific binding levels were also high for both AD and control. A higher specific binding was observed in the gray matter of the AD case (around 25–30 fmol/mg) compared to control (around 15–20 fmol/mg), regardless of the blocker used [ASEM or unlabeled KIn-83, both at 10 μM (Figure 6C)].

^125^I-α-bungarotoxin has been suggested as the *in vitro* gold-standard radioligand for α7 nAChR.^19,25^ The α7 nAChRs are widely distributed in the mammalian brain, with highest receptor density in the hippocampus, hypothalamus, amygdala, and cerebral cortex and lowest receptor density in the cerebellum.^26^ The regional binding of ^3^H-KIn-83 was comparable with the pattern of ^125^I-α-bungarotoxin binding earlier demonstrated by Härfstrand et al.,^38^ showing high specific binding in the hippocampus, hypothalamus, amygdala, and the cerebral cortex of the rat brain.^27^ The ^3^H-Kin-83 binding was completely abolished by ASEM, unlabeled KIn-83, and other ASEM derivatives included in the autoradiographic blocking study.

In a previous recent study from Donat and collaborators, it was described that the specific binding of ^125^I-Iodo-ASEM was lower in the rat and mouse brain when compared to ^125^I-α-bungarotoxin.^19^ In the present study, ^3^H-KIn-83 showed a similar binding signal to ^125^I-α-bungarotoxin using a lower concentration of the tracer (0.8 nM vs 1.4 nM, respectively). Although ^125^I-Iodo-ASEM allows sensitive and selective imaging of α7 nAChR *in vitro*, with better signal-to-noise ratio than previous described tracers,^19^ our data suggests that ^3^H-KIn-83 binds to the brain regions of interest at a higher extent, showing a high affinity and becoming a promising more selective target for α7 nAChR.

It is important to notice that when ^3^H-KIn-83 was tested with ARG using the human brain from a single AD case and a cognitive healthy control (Figure 6), a higher specific binding was observed in the gray matter of the AD case. However, the level of nonspecific binding observed in the human brain was relatively high, especially compared with the low levels obtained when using the rat brain. This might be due to the inter-species differences and should be further tested in more detail in order to potentially improve the chemical properties of KIn-83 in order to decrease the possible off-target binding observed in the human brain. A higher binding of ^18^F-ASEM across the brain regions has earlier been observed in the PET studies of patients with mild cognitive impairment (MCI) compared to cognitive intact individuals as a sign for higher availability of a7n-AChR in MCI compared to healthy subjects.^28^

### NHP Brain PET

**Figure 7:**
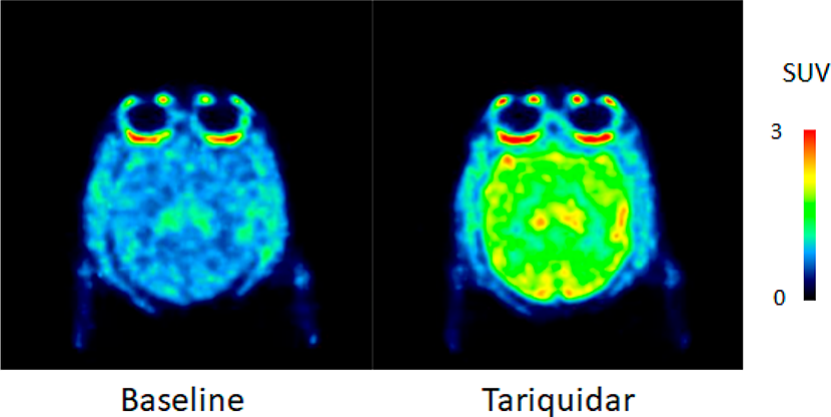
PET summation images of ^11^C-KIn-83 at baseline and after administration of tariquidar.

**Figure 8:**
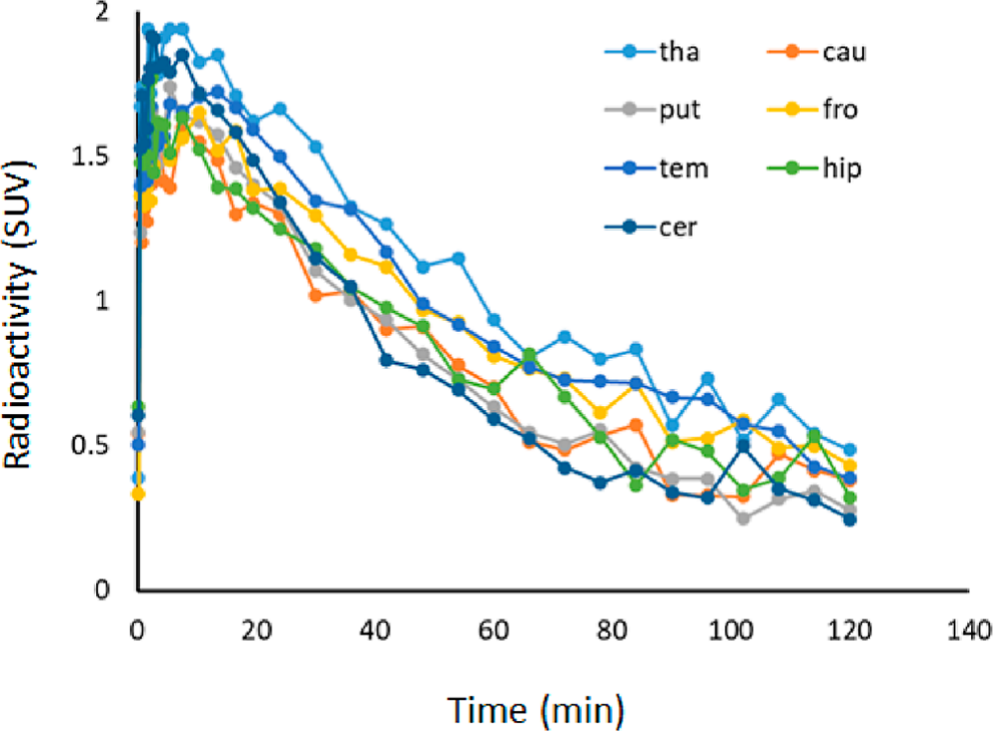
Representative time activity curves of ^11^C-KIn-83 at baseline.

**Figure 9:**
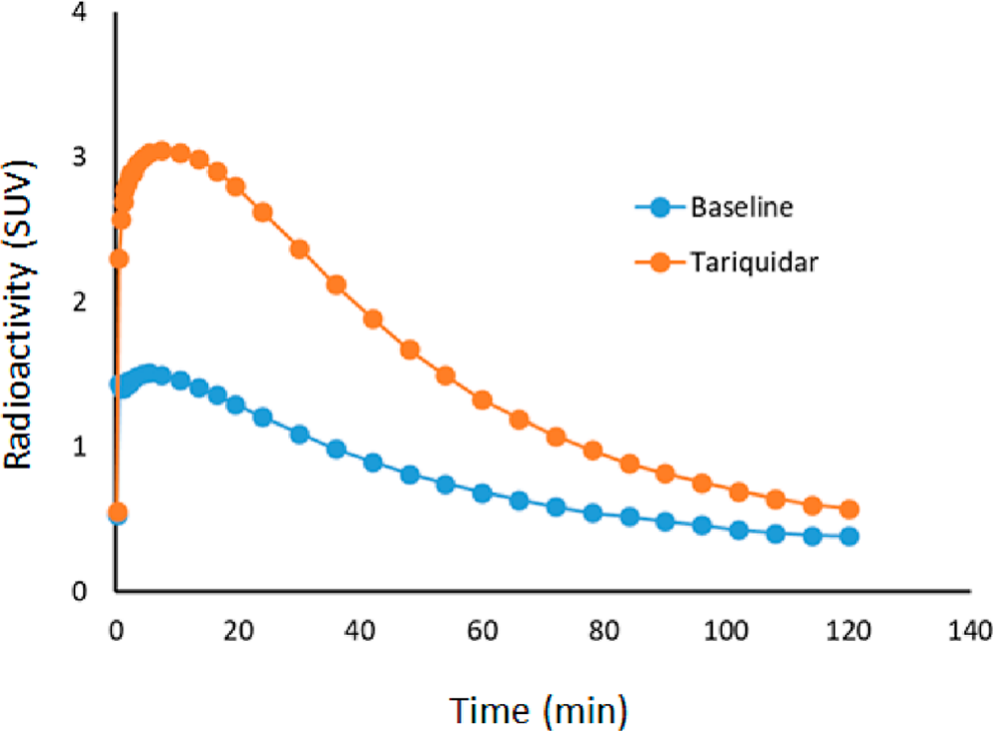
Whole brain time activity curves of ^11^C-KIn-83 at baseline and after administration of tariquidar.

**Figure 10:**
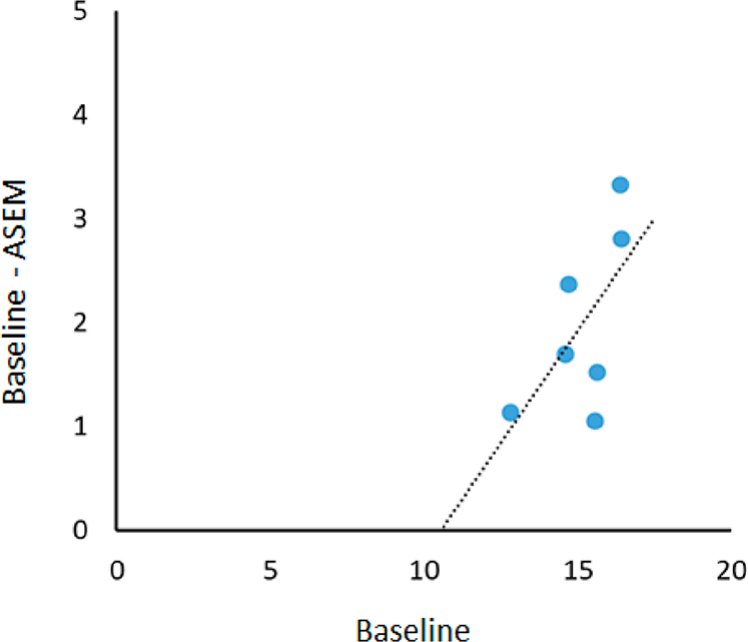
Lassen occupancy plot of ^11^C-KIn-83 by ASEM blocking.

At the time of the injection, the injected radioactivity of ^11^C-KIn-83 was 146 ± 10 MBq, and the injected mass was 6.6 ± 2.6 μg. Images of summated PET are shown in Figure 7. The whole brain uptake of ^11^C-KIn-83 was 1.6 standardized uptake value (SUV) at the peak under the baseline condition. Representative regional time activity curves (TACs) are shown in Figure 8. The uptake of ^11^C-KIn-83 was high in thalamus (1.5 SUV for the total acquisition time), middle in the cortex (1.07–1.17), and low in the basal ganglia and cerebellum (0.99–1.07). The distribution pattern of ^11^C-KIn-83 was similar to other alpha7 nAChR PET ligands such as ^18^F-ASEM.^13^ The brain uptake of ^11^C-KIn-83 was relatively low compared to other PET radioligands, which are commonly used. One possible mechanism is an efflux by the P-gp at the BBB. A clear increase in the brain uptake was observed after administration of tariquidar as 98% increase of average SUV (Figures 7 and 9). This indicates that ^11^C-KIn-83 is a substrate of P-gp^29^ at the BBB. Additionally, the specific binding of ^11^C-KIn-83 to the alpha7 nAChR was estimated using the ASEM blocking and Lassen occupancy plot. V_T_s decreased in all regions after administration of ASEM with the estimated occupancy as 43%, showing similar occupancy values to previous study using ^18^F-ASEM (Figure 10). This indicates that ^11^C-KIn-83 specifically binds to the alpha7 nAChR. Taken together, ^11^C-KIn-83 is a promising PET ligand for the alpha7 nAChR although the brain uptake was relatively low compared to other PET radioligands.

### Radiometabolite Analysis

**Figure 11:**
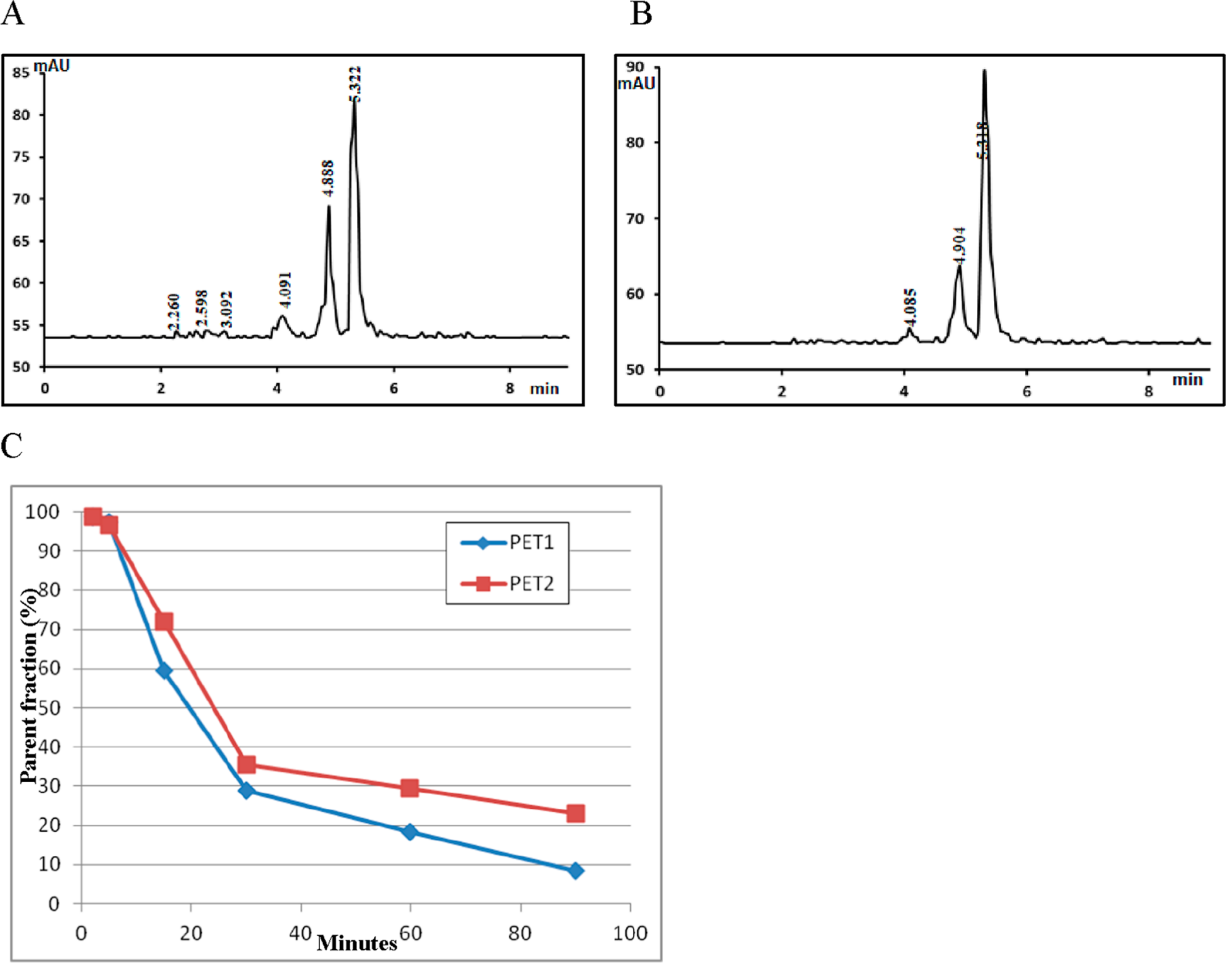
(A) Radiochromatogram of plasma taken 15 min after the injection of ^11^C-KIn-83 under baseline conditions, (B) radiochromatogram of plasma taken 15 min after the injection of ^11^C-KIn-83 after pretreatment with ASEM, (C) *In vivo* metabolism of ^11^C-KIn-83 is shown as the relative plasma composition of the parent compound (PET1: baseline, PET2: after pretreatment with ASEM).

The recovery of radioactivity from plasma into acetonitrile after deproteinization was higher than 95%. HPLC analysis of plasma was carried out following the injection of ^11^C-KIn-83, which eluted at 5.3 min (Figure 11A,B). The parent compound was more abundant at 4 min, representing approximately 96%, and it decreased to <10 at 90 min for PET under baseline conditions (Figure 11C). However, the abundance of the parent compound for PET after pretreatment with ASEM or tariquidar decreased to about 20% (Figure 11C). Two more radiometabolite peaks were observed which were which eluted at 3.9 and 4.6 min (Figure 11A,B). The identity of the radiometabolite ^11^C-KIn-83 was confirmed by co-injection with the non-radioactive KIn-83.

## Materials and Methods

### In Silico Calculations including Machine Learning

Thorough accounts of in silico methodologies applied for PET tracer optimization have been provided earlier.^23^ The final values for binding energies, Ki rates, and residence times of the ASEM analogues are expressed from the free energies computed using the FEP+ utility of Schrödinger software package (Schrödinger Release 2016- 4: LigPrep, Schrödinger, LLC, New York, NY, 2016). The OPLS3 force field was utilized to describe the proteins and ligands. Atomic partial charges for the ligands were computed via the CM1A-BCC algorithm.^30^ The replica algorithm with exchange with solute tempering^31^ was applied using Desmond as the MD engine. The LOMAP mapping algorithm^32^ was applied to set up the calculations and the perturbation pathways. The free-energy calculations were preceded by molecular docking, molecular dynamics, and metadynamic calculations.^22^

Machine learning has been carried out using support vector machine, neural network (NN), and random forest (RF) algorithms. Predictions of the BBB permeation and binding to the P-gp protein of the candidate compounds have therefore been obtained.

### Radiochemistry

#### General

All the precursors (PRE-1 (3-(1,4-diazabicyclo[3.2.2]nonan-4-yl)-6-amino-dibenzo[*b*,*d*] thiophene 5,5-dioxide), PRE-2 (3-(1,4-diazabicyclo[3.2.2]nonan-4-yl)-8-amino-dibenzo[*b*,*d*] thiophene 5,5-dioxide), PRE-3 (3-(1,4-diazabicyclo[3.2.2]nonan-4-yl)-6-hydroxyl-dibenzo[*b*,*d*] thiophene 5,5-dioxide), and PRE-4 (3-(1,4-diazabicyclo[3.2.2]nonan-4-yl)-8-hydroxyl-dibenzo[*b*,*d*] thiophene 5,5-dioxide)) and all the non-radioactive reference standards (KIn-74, KIn-75, KIn-77, KIn-83, KIn-84, and KIn-85) were synthesized by Syngene International, India. All other chemicals and reagents were bought from commercial sources. SPE cartridges SepPak C18 Plus were purchased from Waters (Milford, Mass USA). C-18 Plus cartridge was activated using EtOH (10 mL) and followed by sterile water (10 mL). Liquid chromatographic analysis was performed with a Merck-Hitachi gradient pump and a Merck-Hitachi, L-4000 variable wavelength UV detector. ^3^H-Methyl iodide (^3^H-CH_3_I) was purchased from American Radiolabeled Chemicals (St. Louis, MO, USA).

#### Synthesis of ^11^C-Methyliodide (^11^C-CH_3_I)

[^11^C]Methane ([^11^C]CH_4_) was formed in-target via the ^14^N(p,α)^11^C reaction using nitrogen gas mixed with hydrogen (10%) and 16.4 MeV protons produced by the GEMS PET trace cyclotron (GE, Uppsala, Sweden). The cyclotron target gas was irradiated for 20 min, and a 35 μA beam current was used. ^11^C-Methyl iodide ([^11^C]CH_3_I) was synthesized according to the previously published method.^33^ Target produced [^11^C]CH_4_ was composed in a cooled Porapak Q trap. [^11^C]CH_3_I was released from the trap and subsequently mixed with iodine vapors at 60 °C followed by a radical reaction at 720 °C in a closed circulation system. The produced [^11^C]CH_3_I was collected in a porapak Q trap at room temperature, and the unreacted [^11^C]CH_3_I was recirculated for 3 min. [^11^C]CH_3_I was released from the Porapak Q trap by heating the trap at 180 °C with the flow of helium.

#### General Synthesis of ^11^C-Labeled Compounds

^11^C-Labeled compounds were obtained by trapping ^11^C-CH_3_I at room temperature in a reaction vessel containing the mixture of appropriate precursors and bases in appropriate solvents (Table 1). After the end of trapping, the reaction mixture was heated at ambient temperature. The crude mixture was diluted with sterile water (500 μL) and injected to the built-in HPLC system. The HPLC system was equipped with a semi-preparative reverse phase (RP) ACE column (C18, 10 × 250 mm, 5 μm particle size) and a Merck Hitachi UV detector (λ = 254 nm) (VWR, International, Stockholm, Sweden) in series with a GM-tube (Carroll-Ramsey, Berkley, CA, USA) used for radioactivity detection. A mixture of acetonitrile (40%) and 0.1 M ammonium formate (60%) with a flow rate of 5 mL/min was used as the HPLC isocratic mobile phase, which gave a radioactive fraction corresponding to the desired product with a retention time (*t*_R_) 13–14 min.

#### General Synthesis of ^3^H-Labeled Compounds

The radiosynthesis was performed following the similar procedure described for ^11^C-labeling compounds. ^3^H-CH_3_I was added in the reaction vessel containing the corresponding appropriate precursors 3 (1.0–2.0 mg and 2.7–5.4 μmol), appropriate base in DMF/DMSO (300 μL), and the mixture was sonicated for 15 min. A solution of ^3^H-methyl iodide in toluene (∼1 mCi) was added and then heated at 90 °C for 30 min. 300 μL of water was added. Analysis and purification were performed by LaChrom HPLC on an ACE 5 C18 HL column (250 × 100 mm). The product was eluted with the mobile phase of 40% acetonitrile in ammonium formate (AF, 0.1 M) with a flow rate of 5 mL/min monitored with UV (254 nm) and radioactivity detectors. After repeats of synthesis and combination of collected fractions, solvents in the fraction were removed by SPE, and the product was formulated in ethanol/water. The product ^3^H-KIn74/^3^H-KIn83 was analyzed and identified by HPLC. The retest of radiochemical purity was performed before it was used for the ARG experiment.

#### Isolation of ^11^C/^3^H Labeled KIn-74, KIn-75, KIn-77, KIn-83, KIn-84, and KIn-85

The corresponding radioactive fraction collected from HPLC was diluted with sterile water (50 mL). The resulting mixture was passed through a SepPak tC18 plus cartridge. The cartridge was washed with sterile water (10 mL), and the corresponding isolated ^11^C/^3^H-product was eluted with 1 mL of ethanol into a sterile vial containing PBS (9 mL). The formulated product was then sterile filtered through a Millipore Millex GV filter unit (0.22 μm) for further use.

### Quality Control and MA Determination

The radiochemical purity and stability of ^11^C-KIn-74, ^11^C-KIn-75, ^11^C-KIn-77, ^11^C-KIn-83, ^11^C-KIn-84, and ^11^C-KIn-85 were determined using HPLC equipped with an analytical ACE RP column (C18, 3.9 Ø × 250 mm, 5 μm particle size), Merck-Hitatchi L-7100 Pump, L-7400 UV detector, and GM tube for radioactivity detection (VWR International). The mobile phase CH_3_CN/0.1% TFA with a gradient HPLC (15–90% in 10 min) and a flow rate of 2 mL/min was used to elute the product. The HPLC liquid flow was monitored with an UV absorbance detector (ƛ = 254 nm) coupled to a radioactive detector (b-flow, Beckman, Fullerton, CA). The identity of the radiolabeled compounds was confirmed by HPLC with the co-injection of the corresponding authentic reference standard.

The MA was calculated by analytical HPLC following the method described elsewhere.^34^

### *In Vitro* ARG

#### General

Tissue from the thalamus from an AD patient (86 years old, Braak stage 5, 4 h of *postmortem* delay) and an age-matched cognitive healthy individual (84 years old, 5:35 h of *postmortem* delay) were obtained from the Netherlands Brain Bank (Amsterdam, the Netherlands). Autopsies were executed in a method similar to that defined previously.^35−37^ Cases were neuropathologically confirmed using conventional histopathological stains in fresh frozen tissue.

In case of both human and rat brains, fresh frozen postmortem tissue was sectioned on a cryomicrotome (Leica CM 1860 Leica, Nussloch, Germany), thaw mounted to poly-*l*-lysine-treated glass plates, dried at room temperature, and stored at −20 °C until use. The thickness differed from human tissue (20 μm) and rat tissue (10 μm).

#### *In Vitro* ARG Using ^11^C-Labeled Compounds

For the preliminary screening of compounds, labeling with ^11^C was carried out in rat brain tissue using ARG for testing the potential binding to the target. Slides were thawed at room temperature and pre-incubated in PBS for 10 min following incubation with the labeled compound at 0.01MBq/mL for 30 min. Non-specific binding was determined in the presence of excess of unlabeled reference compounds and/or other ASEM analogues at 10 μM.

After incubation, the slides were washed twice for 3 × 3 min each in ice-cold PBS followed by washing with distilled water. The slides were then dried and exposed to phosphor-imaging plates (Fujifilm Plate BAS-TR2025, Fujifilm, Tokyo, Japan) before scanning in a Fujifilm BAS-5000 phosphor imager (Fujifilm, Tokyo, Japan) at a resolution of 25 μm/pixel. For calibration, 20 μL of aliquots of the incubation solution was dropped onto a filter paper and scanned together with the sections. The sections were analyzed by Multi Gauge 3.2 phosphor imager software (Fujifilm, Tokyo, Japan). The specific binding was defined as subtracting the non-specific binding from the total binding, expressed as percentage of total binding (100%). If the compound did not show specific binding to the brain regions of interest, it was discarded for further analysis.

#### *In Vitro* ARG Using ^3^H-KIn-83

ARG experimental procedures using tritiated compounds were previously described elsewhere^34^ and in brief carried out as follows; slides were thawed at room temperature and incubated with radioligands in binding buffer (50 mM Tris HCl) at the desired concentration (0.8 or 1 nM) for 60 min. The binding was displaced on adjacent sections with the cold compound (unlabeled compound), other ASEM analogues, and ASEM at 10 μM. After incubation, the slides were washed three times in a buffer (50 mM Tris HCl, 120 mM NaCl, 5 mM KCl, 2 mM CaCl_2_, and 1 mM MgCl_2_, at pH 7.4) followed by a brief wash in distilled water. The slides were dried and exposed to new phosphor-imaging plates (Fujifilm Plate BAS-TR2025, Fujifilm, Tokyo, Japan). Tritium micro scale standards (American Radiolabeled Chemicals Inc.) were placed in cassettes together with the sections for calibration and quantification of the binding density.

For the analysis of images, the phosphor-imaging plates were exposed for 90 h. Then, the scanned films were processed in a phosphor imager (Fujifilm BAS-5000, Fujifilm, Tokyo, Japan). Analysis was accomplished using Multi Gauge 3.2 phosphor imager software (Fujifilm, Tokyo, Japan). Manual delineation was performed on each digital image visually using three- to fourfold magnification. Mean pixel values of regions of interest (ROIs) from each section were transformed into radioactivity values using the tritium calibrating standards and recalculated as binding density (fmol/mg protein). Based on these measurements, specific binding values were calculated using the binding values observed in the absence (total binding) or presence (nonspecific binding) of the unlabeled compound. Specific binding was calculated and expressed as percentage of total binding (100%) or fmol/mg.

### Study Design in NHPs, PET Procedure, and Quantification

The study was accepted by the Animal Ethics Committee of the Swedish Animal Welfare Agency (N185/14) and was executed according to “Guidelines for planning, conducting, and documenting experimental research” (Dnr 4820/06–600) of the Karolinska Institutet. The NHPs were kept in the Astrid Fagraeus Laboratory of the Swedish Institute for Infectious Disease Control, Solna, Sweden.

Four cynomolgus monkeys (two females and two males, body weight 4500–8410 g) were used. For three NHPs, brain PET was performed under a baseline condition. For one of these NHPs, the measurement after tariquidar (2.2 mg/kg) administration was also performed. For these three experiments, only venous blood sampling was carried out. Another NHP was measured before and after ASEM (1.24 mg/kg) administration. Arterial blood sampling was performed in this experiment for the measurement of the plasma input function.

Anesthesia was carried out by the intramuscular injection of ketamine hydrochloride (10 mg/kg) at the Astrid Fagraeus Laboratory and maintained by the administration of a mixture of sevoflurane, oxygen, and medical air through endotracheal intubation. The head was halted using a fixation device.^38^ A Bair Hugger model 505 warming unit (Arizant Healthcare, MN) was used to maintain the body temperature and was continuously monitored using an esophageal thermometer. Heart rate, blood pressure, respiratory rate, and oxygen saturation were continuously checked throughout the experiments. Fluid balance was maintained by nonstop infusion of saline.

PET experiments were performed using a high-resolution research tomograph (Siemens molecular imaging).^39^ A 6 min transmission scan using a single ^137^Cs source was carried out before the ^11^C-ligand injection. List mode data were acquired continuously for 123 min (three NHPs) or 93 min (NHP for ASEM administration) immediately after the intravenous injection of the radioligands. Images were reconstructed by the ordinary Poisson-3D-ordered subset expectation maximization (OP-3D-OSEM) algorithm with 10 iterations and 16 subsets including modeling of the point spread function.

The ROIs were delineated manually on MRI images of each NHP for the whole brain, cerebellum, caudate, putamen, thalamus, frontal cortex, temporal cortex, and hippocampus. The summed PET images of the whole duration were co-registered to the MRI image of the individual NHP. After applying the co-registration parameters to the dynamic PET data, the time–activity curves of brain regions were generated for each PET measurement. Average SUV was calculated for each brain regions. For the experiment of ASEM administration, the target occupancy was estimated by the Lassen occupancy plot using *V*_T_ calculated by two tissue compartment using metabolite corrected plasma radioactivity.

### Radiometabolite Analysis

Arterial blood samples (2 mL) were drawn from the monkey at different time points such as 4, 15, 30, 60, and 90 min after the injection of ^11^C-KIn-83. A reverse-phase HPLC method was utilized to determine the percentages of radioactivity corresponding to unchanged ^11^C-KIn-83 and its radioactive metabolites during the course of a PET measurement. Analysis of radiometabolite was carried out according to a method published elsewhere.^40^

## Conclusions

In the present work, an efficient synthesis and screening strategy for six novel ^11^C-labeled ASEM analogues were established, yielding the target compounds. Specific binding in the ARG studies was further studied by ^3^H-KIn-83, which showed the most promising features by the initial ARG screening with six ^11^C-compounds. However, the relatively lower brain uptake *in vivo* evaluation in NHP showed favorable properties for imaging α7-nAChR. In in silico, modeling could largely sustain the properties of the tracers, giving a microscopic explanation of their origin. These results together suggest that ^11^C-KIn-83 may be an improved PET radioligand for further studies in human for the detection of neuronal nAChRs (α7-nAChR).

## References

[ref1] Papke R. L. Merging old and new perspectives on nicotinic acetylcholine receptors. Biochem. Pharmacol. 2014, 89, 1–11. 10.1016/j.bcp.2014.01.029. 24486571 PMC4755309

[ref2] Kabbani N.; Nichols R. A. Beyond the Channel: Metabotropic Signaling by Nicotinic Receptors. Trends Pharmacol. Sci. 2018, 39, 354–366. 10.1016/j.tips.2018.01.002. 29428175

[ref3] Shytle R. D.; Mori T.; Townsend K.; Vendrame M.; Sun N.; Zeng J.; Ehrhart J.; Silver A. A.; Sanberg P. R.; Tan J. Cholinergic modulation of microglial activation by α7 nicotinic receptors. J. Neurochem. 2004, 89, 337–343. 10.1046/j.1471-4159.2004.02347.x. 15056277

[ref4] Suzuki T.; Hide I.; Matsubara A.; Hama C.; Harada K.; Miyano K.; Andrä M.; Matsubayashi H.; Sakai N.; Kohsaka S.; Inoue K.; Nakata Y. Microglial α7 nicotinic acetylcholine receptors drive a phospholipase C/IP3 pathway and modulate the cell activation toward a neuroprotective role. J. Neurosci. Res. 2006, 83, 1461–1470. 10.1002/jnr.20850. 16652343

[ref5] Papouin T.; Dunphy J. M.; Tolman M.; Dineley K. T.; Haydon P. G. Septal Cholinergic Neuromodulation Tunes the Astrocyte-Dependent Gating of Hippocampal NMDA Receptors to Wakefulness. Neuron 2017, 94, 840–854. 10.1016/j.neuron.2017.04.021. 28479102 PMC5484087

[ref6] Coughlin J. M.; Yang T.; Rebman A. W.; Bechtold K. T.; Du Y.; Mathews W. B.; Lesniak W. G.; Mihm E. A.; Frey S. M.; Marshall E. S.; Rosenthal H. B.; Reekie T. A.; Kassiou M.; Dannals R. F.; Soloski M. J.; Aucott J. N.; Pomper M. G. Imaging glial activation in patients with post-treatment Lyme disease symptoms: a pilot study using [11C]DPA-713 PET. J. Neuroinflammation 2018, 15, 346 10.1186/s12974-018-1381-4. 30567544 PMC6299943

[ref7] Coughlin J. M.; Du Y.; Rosenthal H. B.; Slania S.; Koo S. M.; Park A.; Solomon G.; Vranesic M.; Antonsdottir I.; Speck C. L.; Rootes-Murdy K.; Lerner A.; Rowe S. P.; Wang Y.; Lesniak W. G.; Minn I.; Bakker A.; Smith G. S.; Dannals R. F.; Kuwabara H.; Horti A.; Wong D. F.; Pomper M. G. The distribution of the alpha7 nicotinic acetylcholine receptor in healthy aging: An in vivo positron emission tomography study with [18F]ASEM. Neuroimage 2018, 165, 118–124. 10.1016/j.neuroimage.2017.10.009. 28993233 PMC5738927

[ref8] Coughlin J. M.; Rubin L. H.; Du Y.; Rowe S. P.; Crawford J. L.; Rosenthal H. B.; Frey S. M.; Marshall E. S.; Shinehouse L. K.; Chen A.; Speck C. L.; Wang Y.; Lesniak W. G.; Minn I.; Bakker A.; Kamath V.; Smith G. S.; Albert M. S.; Azad B. B.; Dannals R. F.; Horti A.; Wong D. F.; Pomper M. G. High Availability of the α7-Nicotinic Acetylcholine Receptor in Brains of Individuals with Mild Cognitive Impairment: A Pilot Study Using 18F-ASEM PET. J. Nucl. Med. 2020, 61, 423–426. 10.2967/jnumed.119.230979. 31420499 PMC9374031

[ref9] Thomsen M. S.; Zwart R.; Ursu D.; Jensen M. M.; Pinborg L. H.; Gilmour G.; Wu J.; Sher E.; Mikkelsen J. D. α7 and β2 Nicotinic Acetylcholine Receptor Subunits Form Heteromeric Receptor Complexes that Are Expressed in the Human Cortex and Display Distinct Pharmacological Properties. Plos One 2015, 10, e0130572 10.1371/journal.pone.0130572. 26086615 PMC4472343

[ref10] Wong D. F.; Kuwabara H.; Pomper M.; Holt D. P.; Brasic J. R.; George N.; Frolov B.; Willis W.; Gao Y.; Valentine H.; Nandi A.; Gapasin L.; Dannals R. F.; Horti A. G. Human Brain Imaging of α7 nAChR with [18F]ASEM: a New PET Radiotracer for Neuropsychiatry and Determination of Drug Occupancy. Mol. Imaging Biol. 2014, 16, 730–738. 10.1007/s11307-014-0779-3. 25145965 PMC5344036

[ref11] Halldin C.; Gulyás B.; Langer O.; Farde L. Brain radioligands--state of the art and new trends. Q. J. Nucl. Med. 2001, 45, 139–152. 11476163

[ref12] Halldin C.; Gulyas B.; Farde L. PET studies with carbon-11 radioligands in neuropsychopharmacological drug development. Curr. Pharm. Des. 2001, 7, 1907–1929. 10.2174/1381612013396871. 11772357

[ref13] Hillmer A. T.; Li S.; Zheng M.-Q.; Scheunemann M.; Lin S.-f.; Nabulsi N.; Holden D.; Pracitto R.; Labaree D.; Ropchan J.; Teodoro R.; Deuther-Conrad W.; Esterlis I.; Cosgrove K. P.; Brust P.; Carson R. E.; Huang Y. PET imaging of α7 nicotinic acetylcholine receptors: a comparative study of [18F]ASEM and [18F]DBT-10 in nonhuman primates, and further evaluation of [18F]ASEM in humans. Eur. J. Nucl. Med. Mol. Imag. 2017, 44, 1042–1050. 10.1007/s00259-017-3621-8. PMC5400702 28120003

[ref14] Toyohara J.; Sakata M.; Wu J.; Ishikawa M.; Oda K.; Ishii K.; Iyo M.; Hashimoto K.; Ishiwata K. Preclinical and the first clinical studies on [11C]CHIBA-1001 for mapping α7 nicotinic receptors by positron emission tomography. Ann. Nucl. Med. 2009, 23, 301–309. 10.1007/s12149-009-0240-x. 19337782

[ref15] Horti A. G.; Gao Y.; Kuwabara H.; Wang Y.; Abazyan S.; Yasuda R. P.; Tran T.; Xiao Y.; Sahibzada N.; Holt D. P.; Kellar K. J.; Pletnikov M. V.; Pomper M. G.; Wong D. F.; Dannals R. F. 18F-ASEM, a Radiolabeled Antagonist for Imaging the α7-Nicotinic Acetylcholine Receptor with PET. J. Nucl. Med. 2014, 55, 672–677. 10.2967/jnumed.113.132068. 24556591 PMC4112566

[ref16] Ravert H. T.; Holt D. P.; Gao Y.; Horti A. G.; Dannals R. F. Microwave-assisted radiosynthesis of [18F]ASEM, a radiolabeledα7-nicotinic acetylcholine receptor antagonist. J. Label. Compd. Radiopharm. 2015, 58, 180–182. 10.1002/jlcr.3275. 25720955

[ref17] Vetel S.; Vercouillie J.; Buron F.; Vergote J.; Tauber C.; Busson J.; Chicheri G.; Routier S.; Sérrière S.; Chalon S. Longitudinal PET Imaging of α7 Nicotinic Acetylcholine Receptors with [18F]ASEM in a Rat Model of Parkinson’s Disease. Mol. Imaging Biol. 2020, 22, 348–357. 10.1007/s11307-019-01400-y. 31286348

[ref18] Horti A. G. Development of [ 18 F]ASEM, a specific radiotracer for quantification of the α7-nAChR with positron-emission tomography. Biochem. Pharmacol. 2015, 97, 566–575. 10.1016/j.bcp.2015.07.030. 26232729 PMC4600455

[ref19] Donat C. K.; Hansen H. H.; Hansen H. D.; Mease R. C.; Horti A. G.; Pomper M. G.; L’Estrade E. T.; Herth M. M.; Peters D.; Knudsen G. M.; Mikkelsen J. D. Vitro and In Vivo Characterization of Dibenzothiophene Derivatives [I-125]Iodo-ASEM and [F-18]ASEM as Radiotracers of Homo- and Heteromeric alpha 7 Nicotinic Acetylcholine Receptors. Molecules 2020, 25, 1425 10.3390/molecules25061425. PMC7144377 32245032

[ref20] Wong D. F.; Kuwabara H.; Horti A. G.; Roberts J. M.; Nandi A.; Cascella N.; Brasic J.; Weerts E. M.; Kitzmiller K.; Phan J. A.; Gapasin L.; Sawa A.; Valentine H.; Wand G.; Mishra C.; George N.; McDonald M.; Lesniak W.; Holt D. P.; Azad B. B.; Dannals R. F.; Kem W.; Freedman R.; Gjedde A. Brain PET Imaging of α7-nAChR with [18F]ASEM: Reproducibility, Occupancy, Receptor Density, and Changes in Schizophrenia. Int. J. Neuropsychopharmacol. 2018, 21, 656–667. 10.1093/ijnp/pyy021. 29522184 PMC6030963

[ref21] Wu J.; Liu Q.; Tang P.; Mikkelsen J. D.; Shen J.; Whiteaker P.; Yakel J. L. Heteromeric α7β2 Nicotinic Acetylcholine Receptors in the Brain. Trends Pharmacol. Sci. 2016, 37, 562–574. 10.1016/j.tips.2016.03.005. 27179601 PMC5074342

[ref22] Zhou Y.; Kuang G.; Li J.; Halldin C.; Nordberg A.; Långström B.; Tu Y.; Ågren H. In silico studies of ASEM analogues targeting α7-nAChR and experimental verification. RSC Adv. 2021, 11, 3942–3951. 10.1039/d0ra10435c. PMC9134020 35747361

[ref23] Kuang G.; Zhou Y.; Zou R.; Halldin C.; Nordberg A.; Långström B.; Ågren H.; Tu Y. Characterization of the binding mode of the PET tracer [18F]ASEM to a chimera structure of the α7 nicotinic acetylcholine receptor. RSC Adv. 2017, 7, 19787–19793. 10.1039/c7ra00496f.

[ref24] Zhou Y.; Zou R.; Kuang G.; Långström B.; Halldin C.; Ågren H.; Tu Y. Enhanced Sampling Simulations of Ligand Unbinding Kinetics Controlled by Protein Conformational Changes. J. Chem. Inf. Model. 2019, 59, 3910–3918. 10.1021/acs.jcim.9b00523. 31454236

[ref25] Clarke P. B.; Schwartz R. D.; Paul S. M.; Pert C. B.; Pert A. Nicotinic binding in rat brain: autoradiographic comparison of [3H]acetylcholine, [3H]nicotine, and [125I]-alpha-bungarotoxin. J. Neurosci. 1985, 5, 1307–1315. 10.1523/jneurosci.05-05-01307.1985. 3998824 PMC6565049

[ref26] Baddick C. G.; Marks M. J. An autoradiographic survey of mouse brain nicotinic acetylcholine receptors defined by null mutants. Biochem. Pharmacol. 2011, 82, 828–841. 10.1016/j.bcp.2011.04.019. 21575611 PMC3162045

[ref27] Härfstrand A.; Adem A.; Fuxe K.; Agnati L.; Andersson K.; Nordberg A. Distribution of nicotinic cholinergic receptors in the rat tel- and diencephalon: a quantitative receptor autoradiographical study using [3H]-acetylcholine, [α-125l]bungarotoxin and [3H]nicotine. Acta Physiol. Scand. 1988, 132, 1–14. 10.1111/j.1748-1716.1988.tb08291.x. 3223299

[ref28] Coughlin J. M.; Rubin L. H.; Du Y.; Rowe S. P.; Crawford J. L.; Rosenthal H. B.; Frey S. M.; Marshall E. S.; Shinehouse L. K.; Chen A.; Speck C. L.; Wang Y.; Lesniak W. G.; Minn I.; Bakker A.; Kamath V.; Smith G. S.; Albert M. S.; Azad B. B.; Dannals R. F.; Horti A.; Wong D. F.; Pomper M. G. High Availability of the α7-Nicotinic Acetylcholine Receptor in Brains of Individuals with Mild Cognitive Impairment: A Pilot Study Using 18F-ASEM PET. J. Nucl. Med. 2020, 61, 423–426. 10.2967/jnumed.119.230979. 31420499 PMC9374031

[ref29] Kannan P.; Telu S.; Shukla S.; Ambudkar S. V.; Pike V. W.; Halldin C.; Gottesman M. M.; Innis R. B.; Hall M. D. The ″Specific″ P-Glycoprotein Inhibitor Tariquidar Is Also a Substrate and an Inhibitor for Breast Cancer Resistance Protein (BCRP/ABCG2). ACS Chem. Neurosci. 2011, 2, 82–89. 10.1021/cn100078a. 22778859 PMC3369725

[ref30] Harder E.; Damm W.; Maple J.; Wu C.; Reboul M.; Xiang J. Y.; Wang L.; Lupyan D.; Dahlgren M. K.; Knight J. L.; Kaus J. W.; Cerutti D. S.; Krilov G.; Jorgensen W. L.; Abel R.; Friesner R. A. OPLS3: A Force Field Providing Broad Coverage of Drug-like Small Molecules and Proteins. J. Chem. Theory Comput. 2016, 12, 281–296. 10.1021/acs.jctc.5b00864. 26584231

[ref31] Shivakumar D.; Harder E.; Damm W.; Friesner R. A.; Sherman W. Improving the Prediction of Absolute Solvation Free Energies Using the Next Generation OPLS Force Field. J. Chem. Theory Comput. 2012, 8, 2553–2558. 10.1021/ct300203w. 26592101

[ref32] Liu P.; Kim B.; Friesner R. A.; Berne B. J. Replica exchange with solute tempering: A method for sampling biological systems in explicit water. Proc. Natl. Acad. Sci. U.S.A. 2005, 102, 13749–13754. 10.1073/pnas.0506346102. 16172406 PMC1236566

[ref33] Andersson J.; Truong P.; Halldin C. In-target produced [11C]methane: Increased specific radioactivity. Appl. Radiat. Isot. 2009, 67, 106–110. 10.1016/j.apradiso.2008.09.010. 19013077

[ref34] Jahan M.; Johnström P.; Selvaraju R. K.; Svedberg M.; Winzell M. S.; Bernström J.; Kingston L.; Schou M.; Jia Z.; Skrtic S.; Johansson L.; Korsgren O.; Farde L.; Halldin C.; Eriksson O. The development of a GPR44 targeting radioligand [11C]AZ12204657 for in vivo assessment of beta cell mass. EJNMMI Res. 2018, 8, 113 10.1186/s13550-018-0465-6. 30588560 PMC6306373

[ref35] Hall H.; Halldin C.; Farde L.; Sedvall G. Whole hemisphere autoradiography of the postmortem human brain. Nucl. Med. Biol. 1998, 25, 715–719. 10.1016/s0969-8051(98)00053-5. 9863555

[ref36] Schou M.; Halldin C.; Pike V. W.; Mozley P. D.; Dobson D.; Innis R. B.; Farde L.; Hall H. Post-mortem human brain autoradiography of the norepinephrine transporter using (S,S)-[18F]FMeNER-D2. Eur. Neuropsychopharmacol. 2005, 15, 517–520. 10.1016/j.euroneuro.2005.01.007. 16139169

[ref37] Gillberg P.-G.; Jossan S.; Askmark H.; Aquilonius S. Large-section cryomicrotomy for in vitro receptor autoradiography. J. Pharmacol. Methods 1986, 15, 169–180. 10.1016/0160-5402(86)90065-3. 2422496

[ref38] Karlsson P.; Farde L.; Halldin C.; Swahn C.-G.; Sedvall G.; Foged C.; Hansen K. T.; Skrumsager B. PET examination of [11C]NNC 687 and [11C]NNC 756 as new radioligands for the D1-dopamine receptor. Psychopharmacology 1993, 113, 149–156. 10.1007/bf02245691. 7855175

[ref39] Varrone A.; Sjöholm N.; Eriksson L.; Gulyás B.; Halldin C.; Farde L. Advancement in PET quantification using 3D-OP-OSEM point spread function reconstruction with the HRRT. Eur. J. Nucl. Med. Mol. Imag. 2009, 36, 1639–1650. 10.1007/s00259-009-1156-3. 19437012

[ref40] Moein M. M.; Nakao R.; Amini N.; Abdel-Rehim M.; Schou M.; Halldin C. Sample preparation techniques for radiometabolite analysis of positron emission tomography radioligands; trends, progress, limitations and future prospects. TrAC, Trends Anal. Chem. 2019, 110, 1–7. 10.1016/j.trac.2018.10.019.

