# Correction to “Development of ^11^C-Labeled ASEM Analogues for Detection of Neuronal Nicotinic Acetylcholine Receptors (α7-nAChR)”

**DOI:** 10.1021/acschemneuro.2c00538

**Published:** 2022-09-23

**Authors:** Sangram Nag, Patricia Miranda-Azpiazu, Zhisheng Jia, Prodip Datta, Ryosuke Arakawa, Mohammad Moein, Zhou Yang, Yaoquan Tu, Laetitia Lemoine, Hans Ågren, Agneta Nordberg, Bengt Långström, Christer Halldin

Unfortunately, a column in Table 2 in this article was duplicated. Correct entries in the column with heading KI-n83 are now included in the corrected version of this table.

The text “, and KI-n85” at page 354, left colum, line 9 from below, should be removed. The corrected sentence reads “For the compounds substituted at positions R1 and R2, we can see that when ΔΔ*G* relative to ASEM is greater than 1 kcal/mol (e.g., KI-n74, KI-n75, and KI-n77), the compounds show low inhibition (inhibition <15%).

**Table 2:**
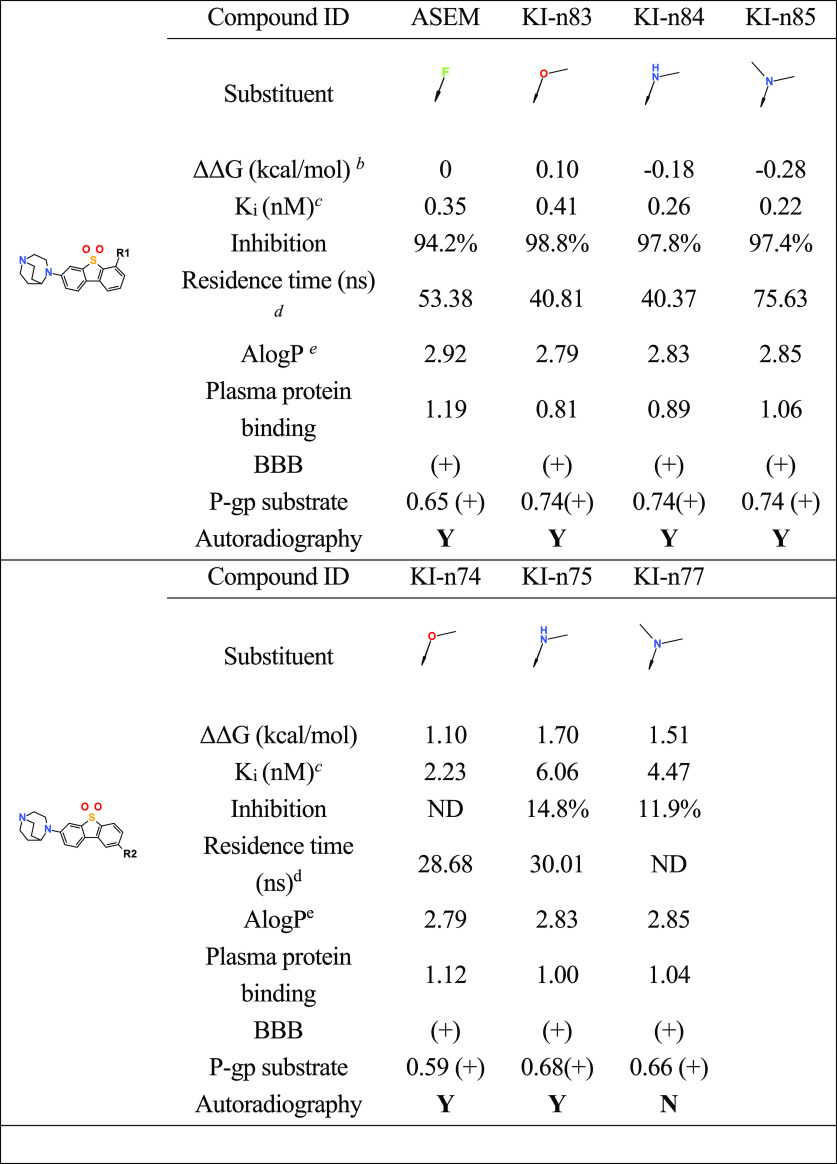
PET Tracer Data for α7-nAChR Using Rational Tracer Design and Machine Learning Methods^a^

a Data for ASEM and Kin85 are excerpts from tabulation given in ref 22.

b (1) The binding free energy is calculated using FEP+ of Schrodinger.^23^ (2) The relative free energy is calculated with ASEM as the reference. Lower is better.^24^

c The *K*_i_ value is calculated using Δ*G* = −*RT* ln(*K*/*K*_0_). The *K*_i_ value of ASEM was reported previously.^20^

d The residence time is calculated with potential scaled MD simulations using Gromacs.

e The physiochemical properties are predicted by machine learning methods based on cheminformatics using Python, sklearn and rdkit.

